# The Use of Intraoperative Neuromonitoring for Cervical Spine Surgery: Indications, Challenges, and Advances

**DOI:** 10.3390/jcm12144652

**Published:** 2023-07-13

**Authors:** John Preston Wilson, Javier Brunet Vallejo, Deepak Kumbhare, Bharat Guthikonda, Stanley Hoang

**Affiliations:** Department of Neurosurgery, Louisiana State University Health Shreveport, Shreveport, LA 71103, USA; jpw002@lsuhs.edu (J.P.W.J.); javier.brunetvallejo@lsuhs.edu (J.B.V.); deepak.kumbhare@lsuhs.edu (D.K.); bharat.guthikonda@lsuhs.edu (B.G.)

**Keywords:** intraoperative neuromonitoring (IONM), cervical spine surgery, cervical deformity, complication avoidance, somatosensory evoked potential (SSEP), transcranial motor evoked potential (TcMEP), machine learning (ML) in IONM, standardization of IONM protocols

## Abstract

Intraoperative neuromonitoring (IONM) has become an indispensable surgical adjunct in cervical spine procedures to minimize surgical complications. Understanding the historical development of IONM, indications for use, associated pitfalls, and recent developments will allow the surgeon to better utilize this important technology. While IONM has shown great promise in procedures for cervical deformity, intradural tumors, or myelopathy, routine use in all cervical spine cases with moderate pathology remains controversial. Pitfalls that need to be addressed include human error, a lack of efficient communication, variable alarm warning criteria, and a non-standardized checklist protocol. As the techniques associated with IONM technology become more robust moving forward, IONM emerges as a crucial solution to updating patient safety protocols.

## 1. Introduction

Spine procedures performed for a range of cervical presentations are becoming more common and are expected to increase each year, with one study predicting a 13.3% increase in surgical volume from 2020 to 2040 for both anterior cervical discectomy and fusion (ACDF) and posterior cervical decompression and fusion (PCDF) procedures [1]. As these procedures become more common, it is paramount to address all areas of improvement, including patient safety and complication avoidance. Neuromonitoring, also known as intraoperative neuromonitoring (IONM), is a vital tool in the operating room. IONM allows for an additional observational window into the status of the patient outside of standard practice anesthesia and vital signs. Monitoring the neurologic properties in real time provides a data-backed representation of the patient’s nervous system’s integrity. Signals produced by IONM can provide guidance to the neurosurgical team throughout the course of the operation and can prevent neurosurgical-related injury by detecting disturbance as potential stressors are introduced [2]. However, although the use of neuromonitoring has become more widespread in recent times, it still faces many challenges. The purpose of this manuscript is to discuss these challenges and potential solutions to improve their utilization. 

This is particularly important for patients undergoing surgical treatment for degenerative cervical pathology, as the information provided by IONM must not be undermined. Ensuring that baseline signals are maintained throughout the operation is of key importance. Careful consideration must be taken when a patient presents with a sudden significant loss in signal amplitude (either SSEP or MEP), a decrease in signal latency (SSEP), or an increase in stimulation strength required to achieve motor response (MEP). While false negatives are not an uncommon occurrence due to any number of extraneous variables, these presentations can be indicative of a decline in the patient’s nervous system integrity. In surgical treatments with cervical decompression or deformity correction, the deviation from IONM signal baselines should not be taken lightly, as iatrogenic damage to this area of the spine can be devastating. Upon confirming that extraneous variables are not affecting the signals, a patient-focused approach to the recovery of baselines should be applied, which can vary from one operation to the next. When applying patient-focused troubleshooting strategies, factors such as the procedure being applied, the progression of the procedure, the state of the patient, and the difference in signal presentations are important considerations.

## 2. Development of Neuromonitoring for Cervical Spine Surgery

IONM was first used in the assessment of nervous system integrity for spine procedures in the 1970s, primarily for scoliosis correction [3]. Shortly thereafter, in 1990, the American Society of Neurophysiological Monitoring was founded with the purpose of investigating and improving the technology. Historically, it has been recognized that IONM is a useful adjuvant to spinal procedures, especially those involving high degrees of surgical manipulation, such as in corrective procedures for cervical deformity or myelopathy [4]. However, for more routine procedures such as disc herniation or moderate cervical stenoses, its routine use is still controversial.

### 2.1. Modalities Used in Neuromonitoring and Advantages of Multimodality Monitoring

A range of modalities is used during spinal surgeries, depending on the surgeon’s preference, the location of the surgical field, or approaches to pathology. Common modalities include somatosensory evoked potentials (SSEPs), transcranial motor evoked potentials (TcMEP), spontaneous and triggered electromyography (sEMG, tEMG), and spinal direct stimulation (D-wave) [5]. Table 1 presents a list of common spine procedures for treating various pathologies, along with their associated IONM modalities. 

The SSEP modality involves stimulation of nerves in the upper and lower extremities. Commonly stimulated sites of the upper extremity include the median and ulnar nerves, while those of the lower extremities involve the peroneal and posterior tibial nerves [6]. Leads placed along the sensory strip of the cerebral cortex will detect peripheral nerve fiber stimulation. SSEP is especially useful in posterior approaches to the spinal column due to the anatomical relationship between the dorsal column of the spinal cord and the application of surgical instruments directly above the spinal pathway [7]. Additional benefits of SSEP in monitoring nerve function include the maintenance of resiliency to anesthetic inhalants or intravenous drugs. 

Motor evoked potentials (MEP) are waveform signal data that reflect the status of descending pathways in the spinal column. By eliciting either transcranial nerve stimulus impulses to the motor strip or stimulation peripherally directly to the muscles, MEP allows the surgeons to monitor signal generation in the upper or lower extremities. Anterior approaches to the spinal column receive great benefit from this modality, in large part due to the anatomical relationship between the ventral nerve roots and lateral motor pathways in the spinal cord [7]. MEPs are utilized in large part to ensure a consistent baseline when manipulations are applied to the spine, such as during the placement of instrumentation, tumor resection, or deformity correction. Criticism of MEP stems from its low specificity, as there can be a high rate of false positives due to the use of anesthetics or paralytics. Taking these considerations into account, MEP remains the most reliable modality for detecting neurologic deficits. 

Free-running EMG holds relevance in its ability to continuously monitor nervous system disturbances. EMG, while not a novel modality, does hold significance in its ability to serve as a baseline reference prior to surgical maneuvers or manipulation by the surgeon. The application of SSEP (ascending pathway functionality), MEP (descending pathway functionality), and EMG (nervous system disruption) when observed together allows for observation of comprehensive nervous system integrity. While each modality has its own set of weaknesses when used in isolation, the simultaneous evaluation of these waveform signals with additional surgical data input provides a clinical picture that can be used to determine any significant disruption. Nevertheless, the use of IONM has not been widely adopted as standard practice, and there are ongoing debates about which modalities should be used for specific spine procedures. Moreover, varying patterns in signal detection for various types of nervous system disturbances have resulted in false-positive or false-negative results. Several studies have investigated these pitfalls in cervical spine procedures, and current solutions center around the use of multiple modalities to provide as much of a comprehensive picture as possible [8]. 

Another area of active research is centered around the optimization of stimulation leads applied to the patient. The first electrodes used for IONM were direct needle leads in the 1960s. Since then, there have been new electrodes available: paddle and percutaneous leads in the 1980s, tined leads in the 1990s, multi-column electrodes, or “lead arrays” in the 2000s, and most recently, high-density electrode arrays [9]. The use of high-density electrode arrays improves signal processing by decreasing the distance between grouped electrodes, allowing for more electrodes to be implemented in a confined space, and increasing the ability to better detect neurophysiologic signals [10]. Questions surrounding the stimulation leads offered and which ones to employ are ongoing investigations that will need to be addressed to help standardize their use. 

### 2.2. Strategies for Protocol Standardization 

Significant efforts are being made to standardize protocols and optimize signal processing to improve signal reliability and minimize artifacts. A study investigating whether improved IONM signals correlated with more favorable postoperative outcomes was a retrospective review of 29 patients from 2013 to 2017. In the 22 patients who underwent surgical decompression for cervical myelopathy, the IONM signal was found to be improved in all patients [11]. Signal improvement was defined as increases in TcMEP amplitude and SSEP amplitude, as well as decreases in SSEP latency. Proactive strategies that led to their improvement in IONM data generation took the form of having a highly trained neurophysiologist with a consistent monitoring protocol, stimulus-adjusted amplitudes for each patient, and the utilization of intravenous anesthetic agents compatible with IONM. 

There has also been an emergence in the use of artificial intelligence in spine surgery. Natural language processing (NLP), one form of machine learning (ML) strategy, has been used to identify patients receiving neuromonitoring, performing at an improved rate compared to traditional administrative codes [11]. In addition, deep learning models have been used for training, validation, and testing of IONM signal changes [11]. Moreover, support vector regression models (SVM), another form of machine learning, have been used to predict dynamic SSEP changes due to various artifacts [12]. The model demonstrated the capability to accurately predict dynamic changes in SSEP due to feature artifacts that included heart rate, blood pressure, body temperature, and anesthetic doses. 

**Figure 1:**
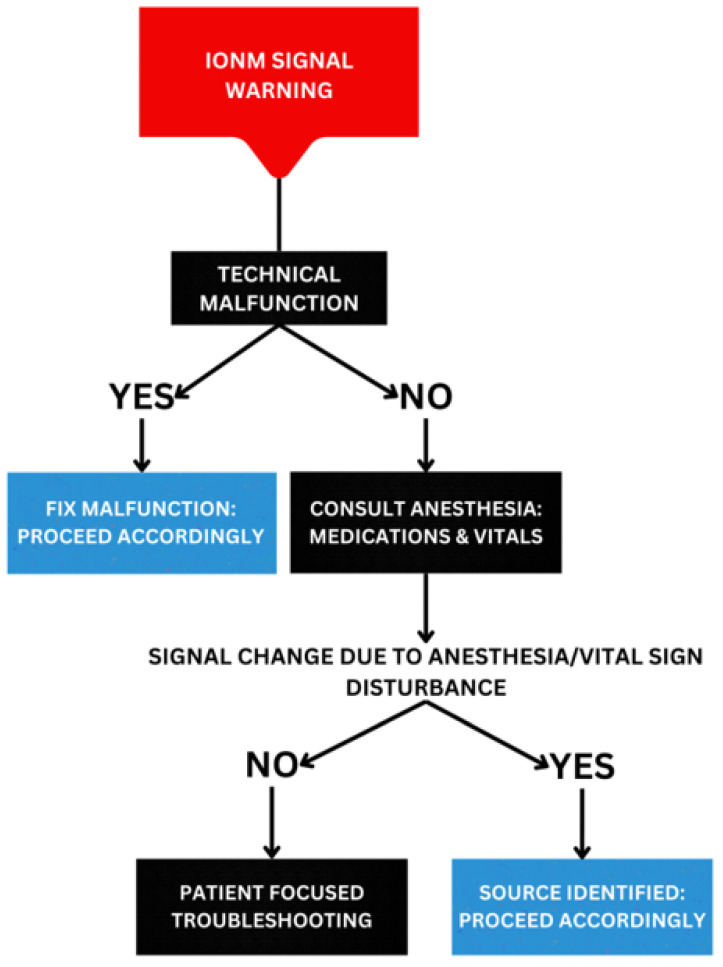
Checklist strategy for approaching IONM signal disturbance-generating alarm criteria. Color of the boxes indicates: red for IONM signal warning, black for further troubleshooting needed, and blue for resolution of disturbance.

Once an IONM change is found, such as the loss of motor potential, a protocol needs to be followed to minimize its impact. In current practice, protocols for checklist strategies addressing IONM signals meeting warning criteria in cervical procedures vary across institutions. Variability arises from differences in IONM staff expertise, surgical staff, and institution-specific guidelines. The checklist approach to IONM signals warranting a response should systematically address potential sources of disturbance. The most easily identifiable source of disturbance is a technical malfunction. As such, the first step in identifying potential disturbances is to verify functional neuromonitoring equipment and correct electrode placement. After confirming equipment functionality, the second step is to consult the anesthesia team to determine if medications or vital sign changes might be causing an IONM signal disturbance. Once surgical team members have discussed these factors, the next step is to shift focus to the patient to identify any active or potential neurological insults. It is important to evaluate the patient’s neck positioning and review recent surgical maneuvers to identify steps that could potentially reverse the damage or prevent further harm. Figure 1 shows a checklist decision tree to identify the source of the IONM disturbance.

### 2.3. Advantages and Disadvantages of Neuromonitoring in Spine Surgery

As the integration of IONM increases, it is important to outline the advantages as well as the associated difficulties. In 2012, the American Academy of Neurology and the American Clinical Neurophysiology Society produced an evidence-based guideline update for the use of IONM in the operating room. Including four class I and eight class II studies, their update supported the proclaimed capabilities of IONM to accurately depict paraparesis, paraplegia, and quadriplegia in patients displaying evoked stimulation changes [13]. The findings in the four class I studies, which comprised 261 patients, revealed that 16–40% of the patients developed events of paraparesis, paraplegia, or quadriplegia. These findings strongly support the foundational use of IONM to detect neurologic injury in the operating room when conducted with a clinical neurophysiologist trained in IONM techniques. 

While IONM allows room for versatility in the application of specific modalities and the location of electrodes, this practice can be seen as a shortcoming due to a lack of standardized protocols. However, the range of multimodal combinations in addition to stimulation points allows for tailoring the IONM protocol for each patient in the setting of abnormal anatomy, preexisting conditions, or tolerance to anesthetics [14]. A study of 209 patients who underwent spine surgery investigated two different individual leg muscles by using muscle motor-evoked potentials for monitoring the tibialis anterior and the abductor hallucis [15]. Although their findings showed that signal change and prognostic values could vary greatly by the muscle type recorded, their study showed that the tibialis anterior muscle showed greater sensitivity in detecting perioperative neurologic injury. The study inadvertently showed the ability to use variable stimulation sites with a specific IONM modality. Moreover, the benefit of having multimodal monitoring by using varying combinations of IONM modalities allows certain modalities to cover the gaps in settings where a different modality being implemented may be affected by any number of intraoperative artifacts. SSEP has the benefit of continuous monitoring throughout the surgery, while also maintaining resistance to disturbance due to inhalational anesthetics, where other modalities such as MEP are more sensitive [16]. On the contrary, TcMEP allows monitoring of the entirety of the motor pathway but lacks continuous monitoring [17]. 

Addressing the difficulties associated with IONM is necessary to properly evaluate its applicability, as surgeons consider its usefulness for each patient. One factor that needs to be considered is the cost associated with IONM utilization. A study investigated 249 patients undergoing Anterior Cervical Discectomy and Fusion (ACDF) procedures, with 48 patients without IONM and 201 patients with IONM. It was found that IONM did not confer a significant reduction in neurological injury between the two groups [18]. Additionally, it was found that the average cost to use IONM was USD 6500 for each patient, which represented a significant financial burden. Other studies questioning the proclaimed benefits of IONM posit that there is no set guideline or requirement for use on every spine procedure. A 2022 survey presented 20 different surgical scenarios to 193 surgeons to assess which modalities they would use. The findings showed that one main reason for implementing IONM was for medical and legal reasons rather than for complication avoidance [19]. This suggests that IONM utilization can be influenced by the need for defense against malpractice lawsuits.

Criticisms surrounding the alert criteria for signal disturbance thresholds have also been a point of controversy. While an inexperienced IONM operator or surgeon may not recognize signal disturbances attributed to developing neurologic injury when referencing a certain threshold marker, an experienced IONM staff member or surgeon more familiar with the technology may have IONM threshold settings and protocols better suited to identifying developing neurologic damage. This pitfall could also be attributed to the large number of false positives that are usually associated with the use of IONM. A retrospective cohort study of 207 patients undergoing cervical spine surgery divided the cases based on their signal changes, with one group experiencing transient changes in SSEP or MEP, another group with sustained changes in either modality, or the last group with sustained changes in both modalities. While fifty-two patients (25%) were initially considered to have experienced signal drops, fifty of these fifty-two cases (96%) were eventually recognized as false positives [8]. This argues for the need to standardize signal loss thresholds representative of true damage. 

### 2.4. Adoption of Neuromonitoring for Cervical Spine Surgeries

The case for IONM and its role in reducing iatrogenic neurological injury in spine surgery is becoming more widely accepted in recent years, with one study identifying an almost 300% increase in utilization from 2008 to 2014 [20]. IONM is a favorable adjunct, especially in the cervical spine, where there is an increased risk due to the nature of sensitive anatomy, coupled with the need to maintain proper positioning throughout the operation [21]. Surgeons performing procedures in the anterior cervical spine are strong proponents of IONM implementation. One internal database review that investigated anterior cervical discectomy and fusion (ACDF), anterior cervical corpectomy and fusion (ACCF), cervical disc replacement (CDR), and anterior discectomy from 2009 to 2011 found that 8854 of these procedures utilized at least one IONM modality [22]. 

Equally important as the preservation of the anatomy in the cervical spine is the proper positioning of the patient, aiming to prevent any potential iatrogenic injury that may occur due to nerve root compression or nerve traction leading to neuropathy. A survey distributed amongst surgeons in the Cervical Spine Research Society found that IONM used for maintaining adequate patient positioning is one of the more common reasons used in surgery for patients with radiculopathy and myelopathy [23].

Many surgeons are hesitant to apply IONM techniques due to the lack of standardization in optimal modality combinations and protocols for patient safety [24]. This hesitancy in using IONM, related to patient safety concerns in high-risk patients with preexisting pathology, the nature of the operation, and the experience of the IONM team or surgeon, are considerations worth discussing before implementing IONM. However, emerging research into the use of IONM in the setting of high-risk patients regarding complex spinal deformity surgery is providing useful insight into IONM in these patients. A 2022 study investigating the correction of complex cervical deformities in patients from 2000 to 2010 used IONM as a preventive strategy. Investigating the different approaches using an anterior osteotomy versus a traditional posterior osteotomy found no IONM changes in either group [25]. Additional support for successful implementation of IONM as an adjunct to complication avoidance in this study is provided by their finding that anterior osteotomy provides adequate angular corrections for high-grade cervical deformity without neurological complications. 

## 3. Application of Neuromonitoring for Cervical Deformity Surgeries

Cervical spine deformity is generally defined as the loss of a normal, neutral range of the physiological alignment of the cervical spine. Several studies have observed the cervical curvature and alignment of asymptomatic subjects. Although there are slight variations in sagittal measurements, a healthy cervical spine ultimately maintains a proper center of mass for the head and preserves horizontal gaze. 

### 3.1. Etiology of Cervical Deformity 

Given the biomechanics of the cervical spine, any change in tension segments and load bearing can cause cantilever adjustments throughout an interconnected spine. These adjustments, however, are biomechanically distinct in the cervical spine as compared to the thoracolumbar segments. As opposed to the lumbar spine, in which the anterior column transmits around 80% of the load and the posterior column transmits around 20%, the cervical spine distributes force in an opposite manner, with the anterior and posterior columns distributing about 36% and 64% of the force, respectively [20]. Understanding the cervical spine’s biomechanical dependence on posterior elements gives critical insights into the etiology of most cervical deformity cases. 

The majority of cervical deformities can be attributed to iatrogenic causes. Posterior surgical options that interrupt paraspinal musculature can cause progressive atrophy of paraspinal muscles. Surgical interruption of posterior elements, including ligamentous structures, has been associated with an increased risk of postoperative kyphosis. Progressive cervical kyphosis and deformity along the sagittal plane can lead to functional and neurological sequelae. Pain and the inability to maintain a horizontal gaze are common symptoms that may warrant surgical intervention if conservative management fails. The presence of neurological sequelae and specific radiographic findings alone can be enough to warrant surgical management in these cases. 

### 3.2. Utility of Neuromonitoring in Cervical Deformity Surgery 

In light of the diversity of cervical deformity morphologies and surgical options, the implementation of systematic classifications has aided in tailoring surgical management to achieve a greater degree of deformity correction. A systematic review of the literature looking at the outcomes of cervical deformity surgery showed significant improvement in functional outcomes, regardless of the surgical approach [26]. Nonetheless, there is a wide range of major complications associated with deformity surgery, with a study showing rates of major complications ranging from 3% to 44%, with neurological complications averaging 13.5%. Because of the high rate of complications, neuromonitoring is considered a requisite in deformity correction to avoid catastrophic neurological deficits. 

Severe adult cervical deformities where a posterior three-column osteotomy is warranted are associated with high rates of neurological complications. Successful utilization of IONM could offer valuable predictive models for postoperative outcomes regarding new neurological weakness [27]. The authors, performing a retrospective review of IONM used for this purpose, investigated the outcomes from an individual surgeon’s experience. Identification of 56 cases using pedicle subtraction osteotomy for one set of patients and vertebral column resection for another group found that IONM was useful for identifying inflicted neurological deficits with high rates of specificity at varying threshold markers [28]. Interestingly, identification of the variable thresholds set at any threshold, 50%, 75%, or no return to baseline, found accuracy performance rates of 73.2%, 76.8%, 73.2%, and 77.8%, respectively. Therefore, the need to optimize the alert thresholds for improved sensitivity is a major consideration that needs further investigation in all procedures utilizing IONM. 

In terms of the surgical techniques for correction of deformity, there is ongoing controversy regarding which correction technique offers the most favorable surgical outcomes. A study aimed to establish optimal surgical correction technique using anterior or combined anterior and posterior procedures included 243 cases. There was a high rate of complication, as 99 patients, or 40.7%, experienced difficulties with implant displacement, graft dislodgment, pseudoarthrosis, dysphagia, voice hoarseness, wound infection, dural tear, pneumonia, neurological deficits (e.g., quadriparesis, radiculopathy, and C5 root palsy), and injury to the vertebral artery [29]. However, regardless of the surgical treatment used in practice, the importance of proper utilization of SSEP, TcMEP, and EMG to achieve cervical decompression and deformity correction was highlighted. Speculation around the causative factors resulting in the high rates of complications not preventable by IONM could include poorly trained IONM staff or improper stimulation protocols to effectively monitor the neurologic integrity. While there are a range of complications surrounding complex spinal procedures and pathophysiology leading to irreversible damage, there is significant support for the use of IONM utilization to identify changes in a timely fashion. 

## 4. Application of Neuromonitoring for Surgeries for Degenerative Disease 

### 4.1. IONM for Patient Positioning

In addition to the value provided during the intraoperative phase of surgical treatment, IONM also has benefits in ensuring optimal neck positioning to prevent spinal cord injury. Positioning the neck for procedures such as ACDF and treatment for cervical kyphosis is extremely important. One case report outlining the importance of neck positioning was highlighted in an ACDF procedure in a 63 year old man, where upon exposure of the lesioned segment there was a complete loss of SSEP and MEP [30]. Once a new, smaller shoulder pad was introduced to the patient, there was a gradual recovery to 75–80% of baselines. Thus, the surgeon was able to identify IONM changes due to positioning after ruling out other technical errors. This case underscores the importance of IONM technology development in allowing for greater detection of iatrogenic neurological damage. Had this case been done without IONM, a neurological deficit would not have been discovered until after procedural completion. It is also important to establish reliable preoperative IONM baselines prior to surgical manipulation, as spinal cord injury due to improper neck positioning may not be apparent immediately and can develop over the course of a procedure.

### 4.2. IONM for Cervical Myelopathy

Degenerative cervical compressive myelopathy (DCCM) is a degenerative spine disease characterized by compressive symptoms due to narrowing of the spinal canal from nontraumatic causes [31]. Neurologic symptoms due to degenerative spinal disease are recognized as one of the leading causative factors globally and the main risk factor for elderly patients [32]. IONM has been shown to serve as a useful predictor for surgical outcomes with magnetic resonance imaging (MRI) in these patients, in addition to real-time monitoring during the procedures [33]. Soda et al. showed that IONM, in addition to helping with complication avoidance during cervical laminectomy, can also be used as a perioperative predictor for patient outcomes. 

### 4.3. IONM during Anterior Cervical Discectomy and Fusion (ACDF)

ACDF remains a controversial procedure in regards to the need for IONM. However, there has been a growing trend in IONM techniques used in elective ACDF. One study performing an analysis of ACDF in 141,000 patients from 2009 to 2013 found that only 6.8% of cases used IONM [24]. A similar study analyzing 15,395 patients around the same study period of 2007–2014 found IONM was used in 17.1% of cases [34]. The discrepancy between these two studies could be due to various factors, such as the region of the cases included in the study, an academic or private neurosurgical clinic, or other unaccounted-for variables during data consolidation. However, a shared trend between both studies was the use of multimodal IONM techniques, including combinations of SSEP, TcMEP, and EMG. 

An earlier institutional study of 119 patients using SSEP and TcMEP for detection of neurophysiologic alerts found that these modalities were effective in active monitoring of the spinal cord and additionally in predicting patient outcome following the procedure [35]. Out of the one-hundred and nineteen patients included in the study, six raised IONM alerts that prompted action to be taken by the anesthesia or surgical personnel on the case. Postoperatively, three patients had developed new motor weakness. While one patient’s symptoms were accurately predicted by the loss of TcMEP, the other two patients had anesthesia related artifacts suppressing the establishment of true TcMEP baselines. This finding emphasizes the importance of accurately establishing baseline signals in patients prior to surgery. However, establishing a reliable baseline can be difficult for patients with pre-existing myelopathy. One study investigating a group of 88 patients undergoing surgery for cervical myelopathy aimed to identify promising strategies to establish reliable TcMEP baselines. It was found that increasing the stimulation intensity to a higher threshold that did not introduce neurologic damage could help achieve a reliable baseline [36]. Thus, the discovery of protocols to help achieve consistent IONM results is necessary for future research, especially in patients with pre-existing cervical deficits.

In terms of the reliability of different modalities, one study of 427 patients from 1999 to 2001 investigated the role of SSEP as related to false-negative results. It was found that ten patients prompted alerts intraoperatively, with two of these patients waking up from surgery with new motor deficits [37]. TcMEP detected the developing injury with 100% specificity and 100% sensitivity, while SSEP signal monitoring showed a specificity of 25% and a sensitivity of 100%. This finding is not uncommon with current IONM techniques, as there is pushback regarding unnecessary patient billing for sub-optimal modalities in certain procedures. A 2021 study of 249 patients undergoing ACDF procedures included two study groups: one group of 48 non-monitored control subjects and the other group of 201 patients receiving IONM. Their results showed that there was no reduction in development of new neurological deficits in the IONM group and suggested a lack of need for the implementation of IONM in ACDF procedures [18]. 

### 4.4. IONM for Posterior Approaches to Cervical Myelopathy

As with ACDF, posterior approaches to cervical myelopathy have been associated with extensive discussion on the utility of IONM. Posterior approaches to cervical myelopathy often include procedures such as cervical laminoplasty or posterior cervical laminectomy and fusion. While these approaches invoke different solutions to treat the patient’s myelopathy, shared among them are their associated risks and complications. Long-standing applications of IONM in cervical laminoplasty and posterior cervical laminectomy and fusion have been implemented since the early 2000s [38]. Common complications for both of these procedures include spinal cord injury, cervical kyphosis, and implant failure [39]. Although cervical laminoplasty has been associated with lower rates of postoperative complications, proper utilization of IONM techniques could decrease the incidence of unfavorable outcomes. 

Some studies have argued that there is little to gain from the application of IONM in posterior cervical procedures [40]. One study of 498 patients analyzed the accuracy of IONM in predicting new neurological deficits. Of the patients included in the study, all of whom received SSEP, only 121 received both SSEP and MEP monitoring during their procedures [41]. Of the patients included, 21 (4.2%) presented with new neurological deficits. The study found unfavorable positive and negative predictive values for both modalities. However, when analyzing studies arguing against the efficacy of IONM in posterior cervical procedures, it is important to note whether a single modality or multiple modalities of IONM were used since multimodal monitoring is necessary to ensure adequate representation of the entirety of the neurological system. When applying proper IONM protocols and troubleshooting strategies, different results indicating more favorable detection of neurological changes can be observed. For example, in another study investigating 131 cervical laminoplasty procedures, all of which implemented multimodality IONM, alerts were found to be associated with 100% sensitivity and 98.4% specificity [42]. Another systematic review of publications investigating incidences of C5 palsy, a serious complication associated with cervical decompressions, found that IONM was one of the main methods used to detect the neurological deficit [43].

Anticipating the development of more robust systems for IONM modalities capable of interpreting signal waveforms, analyzing anesthesia data, and containing a functional technical detection component, the utilization of multimodality protocols is highly important. In the current era, the implementation of IONM can be highly variable in its success in detecting neurological deficits. However, variability in success can be minimized with an informed surgical staff, proper troubleshooting techniques, and standardization of how signal alerts are managed.

## 5. Patient-Focused Troubleshooting for Cervical Deformity and Decompressive Procedures

Properly managing IONM signal alerts begins with the optimal setting of thresholds for the various modalities, especially with regards to amplitude, latency, or applied voltage. According to the American Society of Neurophysiological Monitoring (ASNM), warning criteria should be tailored to each patient and account for the circumstances of the case. They recommend documenting complete loss of MEP waveforms as major identifiers for warning criteria while also recognizing significant amplitude reduction as an additional minor warning criterion. Current guidelines for general amplitude reduction for both SSEP and MEP recommend setting warning criteria thresholds at 50% and 50–100%, respectively. The variability and subjective nature of these thresholds can be attributed to loosely set guidelines and subjective interpretation of signal waveforms when accounting for outside influences. Nevertheless, success has been found when there is thorough planning for optimal sensitivity and specificity of different alert criteria. 

Optimal management of signal drops in IONM modalities in cervical deformity procedures carries great significance in ensuring favorable surgical outcomes. Poorly managed troubleshooting strategies, especially when considering the additional complications potentially incurred with such strategies, can be detrimental to patient outcomes. The absence of widely adopted procedure-specific strategies has led to variability in how signal drops are approached. For example, in surgical procedures for scoliosis, the Scoliosis Research Society (SRS) found responses to signal drops varied greatly. Analyzing the responses of 205 surgeons, the SRS found reactions to these signal drops included performing a reversal of the attempted correction, attempting the correction again, or aborting the procedure entirely [44]. Nevertheless, it should be noted that when the signal drops are due to vascular compromise leading to spinal cord ischemia as a result of the initial surgical maneuvers, the neurologic deficits may not be reversible [45]. 

Considering the array of difficulties and potential complications that are introduced with the correction of cervical deformity, general guidelines on the next initial steps can be considered. Upon following the preliminary steps to rule out monitoring equipment malfunction and anesthesia-induced changes to IONM signals, the surgeon can begin to assess the most logical steps to be taken without introducing increased risk to the patient. In large part, the logic-based decision-making will be dictated by the surgeon’s progression during the procedure. The presentation of a signal drop after manipulating the cervical spine to correct a deformity requires a different approach than when a signal drop follows the placement of instrumentation. Specifically, if a signal drop happens after the patient has been turned prone, the patient should be placed back in the supine position. If the signal changes after the placement of instrumentation, fluoroscopy should be performed to check the location of the instrumentation with either revision or removal of the instrumentation as indicated. Should a signal change take place following an osteotomy, imaging should be done to check for worsening kyphosis, and further decompression may need to be performed. In situations where manual manipulation of the cervical spine to correct the deformity leads to an immediate drop in SSEP or MEP amplitudes, reversal of the previously taken steps could decompress an impinged vessel or nerve and may lead to signal recovery. This can range from extension, flexion, lateral rotation, or combinations of each maneuver to aid in signal regeneration. 

Similarly, as with cervical deformity operations, decompression of the cervical spine from an anterior or posterior approach can present with IONM signal disturbance due to any number of causes. Upon the presentation of IONM signal artifacts, the assessment of previously completed maneuvers by the surgeon at any given point during the operation can provide useful insight into what the next best steps are. Focused strategies for any individual patient can vary, including but not limited to the reversal of recent surgical maneuvers, the removal of previously installed hardware, and the assessment of the position of the cervical spine. Ultimately, the proper assignment of responsibilities and tasks to complete in any IONM troubleshooting strategy depends on preoperative measures that can be taken to optimize the workflow and collaborative efforts of the surgical staff [46]. Timely interventions that lead to favorable outcomes in instances requiring IONM troubleshooting are dependent upon the capabilities of the surgical staff to efficiently identify the causative signal artifact. The benefit of informing the operative team of their responsibilities in the event of IONM troubleshooting could be the difference between a full functional recovery or a loss of neurologic function for a particular patient. 

## 6. Future Directions

The major pitfall in current IONM applications is the variability in signal waveform insults and subsequent troubleshooting protocols after signal disturbances. Extraneous factors affecting IONM signals include technical malfunction, inhaled anesthetics, and other surgical variables [3,47,48]. These artifactual signal disturbances can reduce the specificity of IONM. In addition, the lack of standardization of checklist protocols can impede the timely response to an actual disturbance [49,50,51]. Artificial intelligence and machine learning techniques offer a potential solution to this problem, as they can be used to develop robust systems to analyze IONM signals in real-time and detect patterns related to artifacts or true surgical insults. Analysis of the wide range of cervical spine procedures utilizing IONM also supports the use of multimodal techniques to offer a comprehensive picture of the neurosurgical integrity of the patient. The delicate anatomy of the cervical spine, the difficulty associated with cervical deformity correction techniques, and the pre-existing deficits from cervical myelopathy introduce a higher risk compared to other spinal regions. Nevertheless, by focusing on the importance of optimal communication among surgical team members to ensure efficient checklist strategies for addressing IONM signal disturbances, it is possible to mitigate the risks associated with human error in anesthetic medications, surgical positioning, and surgical techniques. IONM will continue to play a crucial role in the next generation of cervical spine procedures, as it is essential for implementing comprehensive complication avoidance strategies and ensuring the best possible patient outcomes. 

## Figures and Tables

**Table 1:** IONM modalities and their associated techniques, procedural applications, and contraindications.

Modality	Methodology	Surgical Applications	Contraindications/Pitfalls
**SSEP**	Leads placed in the nerves of the UE (Median, Ulnar)/LE (Posterior tibial, Peroneal) Stimulation applied and monitoring of ascending pathways	Posterior approaches Posterior cervical laminoplasties Cervical Spine Corpectomy	Pre-existing neurologic deficit Latency in signal response recording (varies approximately every 2 min, up to 20 min)
**MEP**	Transcranial electrical stimulation of the motor cortex and subsequent recording of myogenic responses Magnetic stimulation subtype for awake procedures	‘Gold standard’ for intraoperative changes Procedures involving isolated motor pathways or nerve roots	Sensitive to anesthetics and neuromuscular blockades High rate of false positives
**EMG**	Direct lead placement into muscle, no stimulation, and continuous monitoring Triggered-EMG subtype: electrical stimulation of instrumentation (tool, screw)	Minimally invasive spine surgery Transpsoas approach Screw placement Decompressions Nerve root monitoring Triggered-EMG: assessment of hardware placement	Nonspecific, sensitive to a wide array of changes due to artifacts
**Multimodal**	Various combinations of modalities Becoming the mainstay in most IONM cases	SSEP, MEP: cervical myelopathy/radiculopathy, scoliosis surgery, and spinal deformity. SSEP, MEP, and EMG: spinal cord neoplasms, extra/intradural pathologies, and posterior occipital-cervical (trauma, tumor lesions, and congenital deformities).	Requires active adjustment and communication between the surgical team and monitoring staff.

## Data Availability

Not applicable.
